# Biochar application to temperate grasslands: challenges and opportunities for delivering multiple ecosystem services

**DOI:** 10.1007/s42773-023-00232-y

**Published:** 2023-06-12

**Authors:** Robert W. Brown, David R. Chadwick, Tom Bott, Helen M. West, Paul Wilson, Genevieve R. Hodgins, Colin E. Snape, Davey L. Jones

**Affiliations:** 1 School of Natural Sciences, Bangor University, Bangor, LL57 2UW Gwynedd UK; 2 School of Biosciences, University of Nottingham, Sutton Bonington, Loughborough, LE12 5RD UK; 3 Department of Chemical and Environmental Engineering, University of Nottingham, Jubilee Campus, Nottingham, NG7 2TU UK; 4 Centre for Sustainable Farming Systems, Food Futures Institute, SoilsWest, Murdoch University, Murdoch, WA 6150 Australia

**Keywords:** Pastureland, Carbon storage, Greenhouse gas emissions, Offsetting

## Abstract

Grasslands (natural, semi-natural and improved) occupy approximately one-third of the terrestrial biosphere and are key for global ecosystem service provision, storing up to 30% of soil organic carbon (SOC). To date, most research on soil carbon (C) sequestration has focused on croplands where the levels of native soil organic matter (SOM) are typically low and significant potential exists to replenish SOM stocks. However, with the renewed push to achieve “net zero” C emissions by 2050, grasslands may offer an additional C store, utilising tools such as biochar. Here, we critically evaluate the potential for biochar as a technology for increasing grassland C stocks, identifying a number of practical, economic, social and legislative challenges that need to be addressed before the widescale adoption of biochar may be achieved. We critically assess the current knowledge within the field of grassland biochar research in the context of ecosystem service provision and provide opinions on the applicability of biochar as an amendment to different types of grassland (improved, semi-improved and unimproved) and the potential effect on ecosystem provision using a range of application techniques in the topsoil and subsoil. We concluded that the key question remains, is it possible for managed grasslands to store more C, without causing a loss in additional ecosystem services? To address this question future research must take a more multidisciplinary and holistic approach when evaluating the potential role of biochar at sequestering C in grasslands to mitigate climate change.

**Graphical Abstract:**

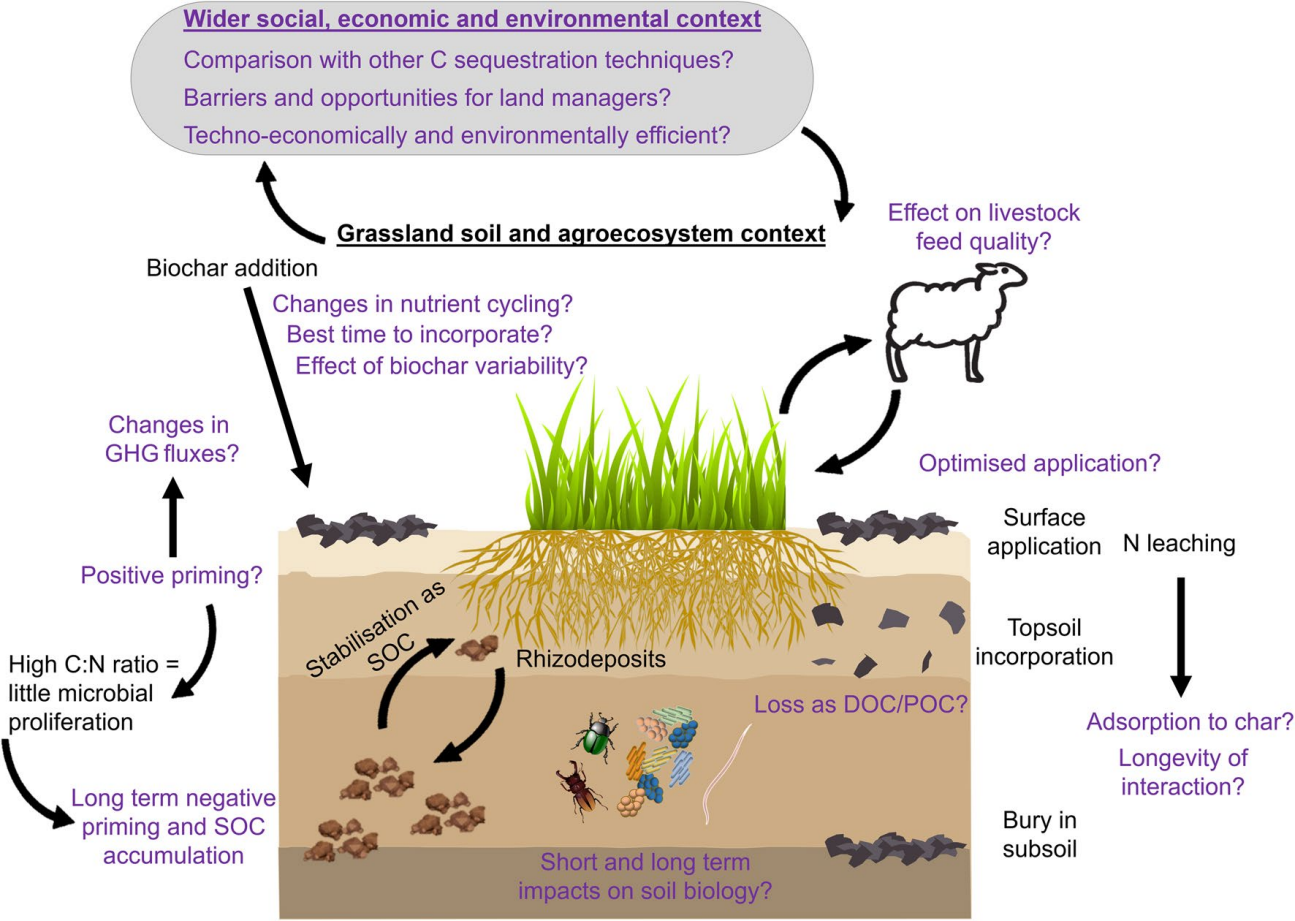

**Supplementary Information:**

The online version contains supplementary material available at 10.1007/s42773-023-00232-y.

## Highlights

- Carbon (C) rich grassland soils may offer an additional C store, utilising tools such as biochar
- Identification of practical, economic, social and legislative challenges for scaled adoption of biochar in grasslands
- Key question: will enhanced C storage in grasslands soils cause a loss in additional ecosystem services?

**Supplementary Information:**

The online version contains supplementary material available at 10.1007/s42773-023-00232-y.

## Introduction

Soil contains large stocks of organic carbon (C), equating to more C than the biosphere and atmosphere combined (Stockmann et al. 2015; le Quéré et al. 2016). Thus, relatively small changes in soil organic carbon (SOC) stocks can have significant impacts on the global C balance, especially the concentration of CO_2_ in the atmosphere. However, anthropogenic agricultural management over the past 10,000 years has reduced soil C stocks by 116 Gt (Amundson and Biardeau 2018; Sanderman et al. 2017). Additionally, it has been suggested that there is further possibility to exploit the soil’s natural C sink potential, which is estimated to be as high as 5.3 Gt of CO_2_-equivalent yr^−1^ (Fuss et al. 2018). Thus, increasing (or restoring) SOC stock is one method that has been proposed to mitigate the effects of climate change (Amelung et al. 2020). Increased SOC stocks are not only beneficial in terms of C sequestration but have also been shown to increase crop yields and improve soil quality (Lal 2016; Oldfield et al. 2019).

For millennia, the act of charring organic material and incorporating it into soil had been recognised as a technique to improve soil fertility and productivity in the Amazon basin (Neves et al. 2004; Lehmann 2009); the *Terra Preta* created and enriched the soil with organic matter as well as other key elements for crop growth (nitrogen (N), phosphorus (P) and potassium (K)) (Chen et al. 2019). In temperate systems, where soils are generally inherently more fertile than tropical systems, char is rarely used as a soil improver. Modern agricultural gains in productivity, driven by the green revolution (increased use of agrochemicals and plant genetic research), reduced the need for organic interventions during the mid to late twentieth century (Evenson and Gollin 2003). However, with increasing interest in restoring anthropogenically degraded soils as well as the increased understanding of anthropogenic climate change and interest in enhanced soil C sequestration, renewed attention in biochar has developed (Kimetu et al. 2008; Lehmann et al. 2006). More recently, the ambition of “net-zero” in the agricultural sector (Reay 2020) and more broadly across society (Deutch 2020) has enhanced the drive to reduce C emissions through sequestration and C offsetting. Several C sequestration technologies have been proposed that may allow managed grassland C stocks to be increased (for example through increased plant species diversity (Chen et al. 2018; Yang et al. 2019), enhanced silicate rock weathering (Gomez-Casanovas et al. 2021; Masiello et al. 2004) or iron (oxyhydr)oxide stabilisation (Wen et al. 2019)), for which further research is encouraged. However, here, we focus specifically on biochar.

A substantial proportion of research has focused on the potential for C sequestration in (arable) croplands, due to their significant C storage potential and the ease in which interventions can be incorporated into normal farming practices (Zomer et al. 2017). However, grasslands (natural, semi-natural and improved) account for around one-third of the terrestrial surface of the planet (Bengtsson et al. 2019), and are globally important in the delivery of many ecosystem services (and associated sustainable development goals). Ecosystem services provided by grasslands are diverse, ranging from provisioning of food, through ruminant livestock production, regulation of climate and water flows, supporting services such as nutrient cycling and pollination, and provision of cultural and aesthetic benefits (Bengtsson et al. 2019; Murray et al. 2013;). In terms of C, grasslands already store significant amounts of SOC (up to 30% of terrestrial SOC; Schuman et al. 2002), due to reduced soil disturbance (i.e., tillage) and high plant C inputs through rhizodeposition and plant litter (Dignac et al. 2017), as well as excreta from grazing livestock (Whitehead 2020). In addition, they have also been shown to be relatively resilient to environmental change (e.g., drought, flooding, warming; Dass et al. 2018). Thus, grasslands play a vital role in soil C sequestration (Bai and Cotrufo 2022). However, grasslands require careful management, as they are frequently grazed and receive additional inputs (including mineral fertilizers and manures), resulting in diffuse losses of nutrients and C to water and emissions of greenhouse gases (GHG) and ammonia to the air (Cai and Akiyama 2017; Hutchings et al. 2007). Modelling has suggested that managed grasslands are largely GHG sources, as opposed to natural grasslands which are C sinks (Chang et al. 2021).

To date, however, little research has been performed on the potential for further C storage in grasslands, particularly examining the role of biochar applications as a C storage strategy with potential added benefits (increased nutrient retention, water storage capacity and plant productivity). This targeted and critical review aims to identify the current gaps in the knowledge, as well as barriers and opportunities regarding biochar application to grassland soils. We collated a number of research questions around the application of biochar to grasslands at the wider scale.

As the pyrolysis product of organic waste, biochar is a chemically stable (~ 2000 years), C-rich material, with its production having the potential to be C neutral or negative (Glaser et al. 2009). Its resistance to microbial decay makes it an ideal candidate for long-term enhanced C storage. Biochar may have beneficial effects on soil quality, adding nutrients (phosphorus (P), sulphur (S) and silicone (Si); Li and Delvaux 2019), buffering pH and reducing bulk density (Alkharabsheh et al. 2021) and N_2_O emissions (Verhoeven et al. 2017), which may subsequently increase grass productivity. Biochar is particularly effective in increasing yields when applied to low fertility or degraded soil, with little or transient effects seen on more fertile or healthier soil (El-Naggar et al. 2019a, b; Jones et al. 2012). Further, while a lack of positive effect is often seen as a negative result in science, this seeming transience of (positive) effect may not be a disadvantage. This is particularly the case if the overall goal of biochar application is focused on C storage alone and not productivity gains, as C storage itself is a beneficial ecosystem service and will contribute to the target of “net-zero” emissions (McLaren et al. 2019; Reay 2020).

However, as biochar encompasses a wide variety of feedstocks and pyrolysis conditions, there is significant variability in biochar quality and physicochemical composition with some biochars being the source of toxic substances (e.g., polycyclic aromatic hydrocarbons (PAHs), volatile organic compounds (VOCs), dioxins and heavy metals) as well as potentially reducing the efficacy of agrochemical and availability of nutrients (Brtnicky et al. 2021; El-Naggar et al. 2019a, b). It should be noted, however, that not all VOCs have negative impacts and some contaminants bound to biochar (e.g., PAHs) are not bioavailable (Brown et al. 2021; Quilliam et al. 2013a, b). The wide diversity in biochar composition makes it difficult to draw generalisations based on individual studies, with biochar properties being the sum of its unique feedstock composition and pyrolysis conditions (temperature and atmosphere composition). Generally, lower temperatures increase its cation and anion exchange capacity (Ferraro et al. 2021), while higher temperature pyrolysis increases both the stability of the biochar and its anion exchange capacity (Banik et al. 2018; Nguyen et al. 2010; Woolf et al. 2021). This allows ‘designer biochar’ properties to be tailored to its application. In an agricultural system this may be either slow release of nutrients, or addition of high stability C to the system for long-term C sequestration.

The status of biochar research in soil, mainly in an arable setting, has been summarised in recent publications by, among others, Joseph et al. (2021), Blanco-Canqui (2021), Brtnicky et al. (2021), and Sun et al. (2021). However, field studies, particularly at large scale, are limited in number, leading to difficulty in drawing robust conclusions (Schmidt et al. 2021; Vijay et al. 2021), with laboratory and mesocosm studies often not being reflective of real-world conditions. Non-significant results are often not published (Amrhein et al. 2019; Lederman and Lederman 2016), potentially leading to publication bias.

## Biochar as a tool for grassland carbon storage

As permanent grasslands (> 5 consecutive years), by their nature, have reduced tillage and disturbance compared to arable systems, they have comparative stability (in both physiochemistry and biology); potentially offering greater persistence and therefore permanence of C storage (Dynarski et al. 2020). Additionally, grassland degradation is also increasing in many parts of the world (Bai et al. 2008), likely leading to a loss in C stocks (Conant et al. 2017). To address this, biochar addition (1 to 50 t C ha^−1^) has been identified as a potential strategy for combatting degradation (i.e., promoting restoration) of grassland, aiding preservation of grassland C stocks and increasing productivity (Bai et al. 2020; van de Voorde et al. 2014; Rafiq et al. 2020). When applied at high rates, this has the potential to double the organic C in the topsoil of many degraded grasslands. However, the addition of biochar to soil has also been shown to illicit a loss of native soil organic matter (SOM) in the short term, through the addition of labile nutrients leading to priming (Cross and Sohi 2011; Wang et al. 2020). Since grasslands are already such a significant store of C, this priming effect may outweigh any benefit of C storage, although there are few long-term field studies (> 10 yr) to critically evaluate the significance of this.

Globally, it has been predicted that biochar production using sustainable feedstocks (e.g., agricultural wastes; crop residues, manures, and biomass crops) and maximising the use of by-products (i.e., bio-oil, syngas and heat) may be able to offset a maximum of 1.8 Pg CO_2_-C_e_ yr^−1^ (~ 12% anthropogenic annual GHG (CO_2_-C_e_) emissions) (Woolf et al. 2010). However, the potential for biochar application as a C storage tool is variable across the world and is generally dependent on the area and availability of applicable land, the current state of soil quality, and level of soil C saturation. For example, as a rough estimate, grasslands (improved and semi-improved) account for ~ 40% of total UK land area (244 000 km^2^; Office for National Statistics, 2015), therefore the maximum technical potential of C removal may be up to 23 Mt CO_2_-e (assuming an optimistic C storage value of 4.8 t CO_2_ per tonne of biochar produced and loading rate of 50 t ha^−1^; Hammond et al. 2011; Roy and Dias 2017). However, the actual (achievable) technical potential is likely to be lower due to a range of social, cultural, legal, economic, and practical barriers.

Regulatory barriers, for example the fact that biochar maybe considered a waste product and therefore might be governed by waste regulation (He et al. 2022; Kane and Ryan 2022), as well as public and stakeholder opinion and potential compensation from C markets (central to the socioeconomic willingness to adopt biochar (Latawiec et al. 2017)), provide challenges and uncertainty in wide-scale adoption.

## State of current biochar grassland research

Biochar research focusing specifically on grassland soils represents a small percentage (~ 2%) of the significant body of research that exists around biochar (> 12,300 results: Web of Science, search string “biochar AND soil” for the period 2006 to 2023). Here, we briefly reviewed the biochar literature in a grassland setting for the most common ecosystem service measurements that were taken in relation to the field study of temperate grasslands. The Web of Science was used as the primary database, utilising the search string "ALL = (biochar AND grassland)". We extracted data over the period 2009 to 2022, producing 206 publications. After excluding non-field-based experiments, as well as metanalyses, and studies not in temperate climates (including Continental, Mediterranean and Oceanic), as well as papers containing no reference to any ecosystem service measurements, a total of 41 papers were taken forward for analysis. These were then examined to identify which ecosystem service parameters were recorded and reported, under the broad themes of (i) provisioning, (ii) regulating, or (iii) supporting and (iv) cultural. Overall, 19 individual ecosystem service indicators were assessed across the themes (Dodd et al. 2023). The full list of papers and ecosystem services measured are summarised in Additional file 1.

Generally, 46% of  the studies reported indicators across at least two of the three ecosystem service themes evaluated here, 10% reported on only one, while 44% included measurements from all three. pH and plant biomass were jointly the most reported ecosystem service indictor measured (54% of studies), followed by some measurement of dissolved nutrients (NO_3_-N, NH_4_-N, PO_4_-P; 46% of studies). However, several ecosystem service indicators are rarely reported, including: porosity (0%), the tea bag index as a metric for C storage potential (0%), earthworm biomass and abundance (0%), percentage plant ground cover (2%) and plant survey (species richness and diversity; 2%). Feedstocks were predominantly wood (58%), or straw- or grass-based (21%).

While few studies on biochar examine  long-term, field-scale effects (Vijay et al. 2021), 67% of the studies last over a year. Equally, the duration of monitoring rarely exceeds 3 years (5%; likely reflecting the nature of scientific funding), however, with the persistence of biochar in soil being estimated at 200–2000 years, this only provides a snapshot of the short-term effects following application. We have little knowledge of the stability of biochar C and its effect on soil health over the long term, particularly in temperate grasslands (in comparison to the study of the legacy effects of *Terra Preta* in the tropics). Equally, the comparative effect of differing feedstock and pyrolysis conditions between biochars is often little explored in terms of creating ‘designer’ biochar for specific grassland applications (Ippolito et al. 2020).

In recent years, there has been an increase in the financial and cultural value put on the environment and the ecosystem services that it provides (Bateman et al. 2019). Thus, metrics of soil and plant health should not be measured in isolation, rather a broad range of measurements across the ecosystem service spectrum allows for a more holistic overview of the benefits (or disadvantages). Biological indicators of soil quality are some of the most responsive to change and of high importance (particularly earthworms as “ecosystem engineers”; Paz-Ferreiro and Fu 2016). The number of species of earthworm is generally higher under grassland compared to arable (Boag et al. 1997; Singh et al. 2021), and while this likely provides increased functional redundancy, the effect of biochar on this diversity and its implications for ecosystem service provision should be explored.

Additionally, grasslands are often hosts to ruminant livestock systems, and while there has been some exploration of effect of biochar in soil on N_2_O emissions from soil, generally showing reductions (Cayuela et al. 2014), ruminant urine patches represent a hotspot of N loading (and subsequent loss) (Chadwick et al. 2018; Marsden et al. 2016). Biochar has previously been explored as a feed amendment for livestock for the abatement of nutrient and GHG losses (Man et al. 2021; Schmidt et al. 2019). While there is some evidence that incorporation of biochar to soil may reduce ruminant urine patch N losses (Mahmud et al. 2018; Taghizadeh-Toosi et al. 2011), the effects of this must be explored on a range of soil types and over time. Equally, the diversity of grassland is rarely reflected in the literature, with the vast majority of studies taking place on lowland agricultural systems, when semi-natural and upland grasslands are also important in extensive livestock systems.

## Potential for net ecosystem service provision change

Over the years, two key themes in soil related biochar research have emerged. These can be broadly characterised into amendment for soil and crop quality improvement (Agegnehu et al. 2017; Jones et al. 2012; Mousavi et al. 2022), and amendment for soil C storage (Chagas et al. 2022; Lehmann et al. 2021; Smith et al. 2016). Few studies have examined the effect on ecosystem multifunctionality (Bolan et al. 2021) i.e., the net effect on ecosystem service provision. Generally, benefits/disadvantages are not isolated and will have interaction effects and feedback mechanisms with other functions and services. As summarised by Blanco-Canqui (2021), the literature indicates that biochar is unlikely to improve all ecosystem services, which is dependent on a plethora of factors (e.g., the type of biochar being used, application rates, and soil properties).

Here, we summarised the potential for biochar as an amendment for grassland, and potential net changes in broad ecosystem service provision in the opinion of the authors of this paper as technical experts in a range of disciplines (biochar, grassland science, soil science, sustainable land use systems, microbial ecology, environmental biology, soil biochemistry, agricultural economics, pyrolysis and fuel science). Authors were asked to score the effects of biochar addition to grasslands on each ecosystem service individually and results were then averaged to produce a final score. The applicability of biochar as a soil amendment in grassland is assessed in Table 1, while the net effect on the potential for ecosystem service provision in improved grassland (determined as the most likely to be amended with biochar) is presented in Table 2. Application of biochar to soil in arable systems was used a comparative reference.

**Table 1:** Assessment of the applicability of biochar as an amendment for grassland

	Practicality of accessing and adding char to the site	Economically viability of the operation	Social and cultural acceptability to farmers and landowners	Effectiveness at C storage	Potential for widescale adoption in the UK	Potential range of biochar addition (t ha^−1^)	Overall score
Arable (for comparison)	9 ± 0.2 (?)	7 ± 0.4 (??)	7 ± 0.4 (??)	7 ± 0.5 (?)	7 ± 0.4 (?)	10–100	7
Improved grassland	7 ± 0.4 (?)	7 ± 0.5 (??)	6 ± 0.4 (??)	6 ± 0.7 (??)	5 ± 0.6 (?)	10–50	6
Semi-improved grassland	5 ± 1.2 (?)	4 ± 1.2 (??)	4 ± 1.1 (??)	5 ± 1.3 (??)	3 ± 0.9 (??)	1–5; 20	4
Unimproved grassland	4 ± 1.1 (??)	3 ± 0.8 (??)	3 ± 1.2 (??)	5 ± 1.7 (??)	2 ± 0.4 (??)	1–10	3

Authors (*n* = 7) scored the applicably of biochar to agricultural land in each category (1 (not-) to 10 (extremely-)) and gave an uncertainty rating for each (??? (very uncertain) to—(certain), the scale being ???, ??, ?, -) and estimated the practical range of biochar that could be added to each land use based on expert opinion. Scores are presented as a mean ± SEM, with the mean uncertainty rating in brackets, rounded to the nearest integer. The potential range of biochar was calculated as the mode of the lowest and highest numbers suggested, respectively (note that the upper potential range of semi-improved grassland was bimodal)

**Table 2:** Assessment of the effect of biochar as an amendment for improved grassland on ecosystem services

Practicality			Relative change from business as usual			
Method of application		Potential/known effect	Provisioning	Regulating	Supporting	Cultural
Topsoil	Surface broadcast (Dry or wetted)	Reduced liming requirement Wind/rain loss Human health (inhalation) Agrochemical adsorption	0 ± 0.9 (??)	1 ± 0.9 (??)	1 ± 0.8 (??)	− 1 ± 1.3 (??)
	Sub-surface application—shallow injection—conventional injection	Reduced liming requirement Agrochemical adsorption	0 ± 0.8 (??)	3 ± 0.4 (??)	2 ± 0.5 (??)	0 ± 0.2 (?)
	Sub-surface application—shallow injection—pneumatic injection	Agrochemical adsorption	0 ± 0.7 (??)	3 ± 0.4 (??)	2 ± 0.5 (??)	0 ± 0.2 (?)
	Mixed with manure / organic resource applications	Increased risk of NH_3_ volatilisation (high manure pH)	1 ± 1.1 (??)	1 ± 0.9 (??)	1 ± 1.2 (??)	-1 ± 0.7 (?)
	Incorporation during reseed	Reduced liming requirement Agrochemical adsorption C priming	1 ± 0.7 (??)	3 ± 0.4 (??)	1 ± 0.9 (??)	0 ± 0.2 (?)
	Slot seeding	Reduced liming requirement	2 ± 0.2 (??)	2 ± 0.2 (??)	2 ± 0.7 (??)	0 ± 0.3 (?)
Subsoil	Trenching/ application to ditch at field boundaries	Agrochemical adsorption	1 ± 1.0 (??)	3 ± 0.5 (??)	1 ± 0.7 (??)	1 ± 1.6 (?)
	Sub-surface application—Deep injection	Unknown	0 ± 0.2 (??)	3 ± 1 (??)	1 ± 0.4 (??)	0 ± 0.0 (?)
	Mole drainage infill	Drain blockage/induces waterlogging	− 1 ± 0.6 (??)	2 ± 0.6 (??)	0 ± 0.7 (???)	0 ± 0.0 (?)
	Surrounding field drains during installation	Unknown	1 ± 1.0 (??)	2 ± 0.6 (??)	1 ± 0.8 (??)	− 1 ± 1.5 (??)

Authors (*n* = 7) scored the likely change on ecosystem services that biochar application will cause, − 10 (extremely negative) to + 10 (extremely positive) with 0 representing no change from business as usual, and gave an uncertainty rating for each (??? (very uncertain) to – (certain), as above). Assessment of ecosystem service categories was based the indicator metrics proposed in Dodd et al. (2023), summarised in Additional file 2: Table S1

Generally, there was consensus that arable cropland remains the most favourable for biochar application (Table 1), likely due to the relative ease of application within normal agricultural practices and the increase in, often depleted, C stocks (Davidson and Ackerman 1993; Paustian et al. 2019). This also suggests that arable soils are more appropriate for the highest potential biochar application rates, with potentially little impact on crop productivity (Jones et al. 2012). However, as discussed above, grasslands occupy a significant amount of land that may be further utilised to sequester C.

Expert opinion suggests that improved grasslands (rather than semi-improved and unimproved grasslands) are likely to be the most favourable for biochar amendments in terms of practicality and economics (Table 2). In contrast, semi-improved and unimproved grasslands are unlikely to be appropriate for biochar application, due to practicality (as, this land is often remote with accessibility issues, resulting in little agricultural traffic, or may be under some form of conservation designation), acceptability and effectiveness at C storage (as C stocks are often high already; Eze et al. 2018). In comparison to improved grasslands, the potential amount of biochar which can be added may also be lower than economically and environmentally viable. There was a perception that considerable uncertainty in understanding the economics and social opinion towards biochar application exists, likely due to a lack of research in these areas (Latawiec et al. 2017).

In terms of potential changes to the provision of ecosystems services, we postulated that there would likely be little substantial change from business as usual using most biochar application methods. There was significant uncertainty associated with most ecosystem service impacts, likely due to the lack of long-term field-scale data, including the practicalities of application, and the fact that few studies have focused on more than two ecosystem services. Topsoil application is likely to be more common than subsoil, due to the relative ease and cost. Arguably surface broadcasting and incorporation during grassland reseeds are likely to be the most common deployment methods, as they can both be done using standard agricultural equipment. However, while injection requires more specialised equipment, it may bring the largest benefits in terms of regulating and supporting services, potentially reducing bulk density and improving water infiltration. It must also be noted that in terms of measurement, reporting and verification (MRV), which will be key to monitoring soil C increases over time, methods that directly incorporate biochar into the soil are likely to be favoured, as they reduce the likelihood that the C is transported/exported through wind and water erosion.

## Future research direction

For almost two decades biochar has been proposed as a potential method of GHG removal and climate change mitigation (Lehmann et al. 2006). However, despite the large number of positive results published on biochar (reviews by Ding et al., (2016), Ali et al., (2017), Liu et al. (2018) and Shaaban et al., (2018)), only a few studies (reviewed recently by Vijay et al., (2021)) have examined the effect of biochar application on soil quality and productivity at the agricultural field scale (Maroušek et al. 2016). This disconnect between research and practice is not surprising, considering the lack of large-scale infrastructure for the production, distribution and application of biochar, and little understanding of the economic and environmental cost (Maroušek et al. 2019). Equally, social research on the opinions of farmers and land managers (who will be key in wide scale adoption of the technology) is extremely scarce (Latawiec et al. 2017). We summarised the key knowledge gaps in biochar research in relation to grassland application by research area, highlighting the key areas for future research and development to underpin wide-scale adoption of biochar amendments in grassland systems:

### Practicality

- What is the best method for field-scale application? In terms of cost to the farmers and ease of adoption, utilisation of current machinery i.e., in isolation or spreading with fertiliser or mixing with livestock (mainly cattle) slurry, is likely to prove the most economically viable option. Equally the interaction effects between biochar, slurry and other organic resources applied to grassland need to be explored.
- When is the best time to incorporate biochar into grasslands, i.e., at reseed (one large loading) or annually (repeated lower loading rates)?
- What is the optimal size of biochar (i.e., chunks/ pellets/ dust) and how does this affect application technology and agroecosystem function?
- Is there a negative priming effect (as grasslands are already large stores of SOC)?  Does this response exhibit in the field over long time periods?
- What is the potential effect on non-CO_2_ GHG (direct N_2_O, indirect N_2_O (NO_3_^−^ leaching, NH_3_ emissions) and CH_4_ efflux) under field conditions?
- Biochar feedstock is often highly variable in its composition (due to being derived from different waste streams and produced under different pyrolysis conditions), so what effect does feedstock variability, type, quality and pyrolysis conditions have on the ability to store C and support ecosystem services in grasslands?
- Where is the feedstock going to come from to make the biochar? This is relevant in areas where there is a spatial disconnect in biochar production and consumption.
- Can we add biochar to the subsoil (e.g. during deep tillage)?
- Is biochar an applicable and/or suitable for C sequestration across a wide range of soil types and pasture types e.g., hay meadows, upland semi-improved and unimproved pastures, some of which may have limits imposed on their management strategies (i.e. environmental stewardship schemes that preclude any inputs)? How spatially variable is the soil’s response to biochar addition?
- Livestock may ingest large amounts of soil (cows up to 1 kg per day (Jurjanz et al. 2012); how would biochar-loaded soil affect the ruminant gut microbiome?
- How does biochar compare with other potential methods to sequester C in agricultural systems, i.e., enhanced silicate rock weathering and iron mediated stabilisation, and could a combination of techniques be appropriate?
- How do we validate the net C gains (e.g. for C accounting purposes)?

### Social

- What are the social and cultural barriers and opportunities for farmers and land managers regarding the use of biochar on grasslands?
- How practical do farmers think it is as a method of C storage, particularly in comparison to other strategies?
- What are the health impacts to grassland farmers handling and spreading biochar?

### Economic

- Is it economically and environmentally efficient to use biochar as a C sequestration technique or do supply chain processes and costs and impacts (i.e., production, transport and application) outweigh the benefits?
- Is on-farm biochar production better than off-site production at a large industrial plant?
- Would agri-environmental scheme payments (i.e., public money) be required to target specific grasslands for biochar application and make this viable? Or, alternatively, how would a just C trading scheme be structured effectively to incentivise farmers to manage land to sequester C.

### Legislative/regulatory

- What are the key waste regulations that would need to be addressed before biochar could be used at scale?
- What is the possibility of application to ‘protected grasslands’ e.g., with conservation designation or in environmental stewardship schemes, measures that preclude inputs to some grassland areas?

## Conclusions

To summarise, biochar has potential as a soil C sequestration tool, adding further benefits to the agroecosystem. Biochar addition into the soil of semi-permanent and permanent grassland systems has been explored to a much lesser extent than in arable cropping systems. This lack of research is hampering the wide-scale adoption of biochar in grasslands. Before national scale policy is developed regarding biochar, much more research is required to holistically assess the impacts on ecosystem service provision as well as the ease of applicability at a field/farm scale, and fully understand the life cycle costs. A key question remains; is it possible for grasslands under management to store more C, without causing a loss in other ecosystem services? This is likely to include the assessment of combinations with other C sequestration techniques (e.g., enhanced silicate rock weathering and Fe mediated stabilisation) to maximise C storage. However, this must be achieved while minimising the negative effects on ecosystem services for example, adding high nutrients to soil may reduce biodiversity with a knock-on effect on pollinators.

## Supplementary Information

**Additional file 1.** List of grassland and biochar papers reviewed to assess the current state of biochar grassland research.

**Additional file 2: Table S1. **Indicator metrics associated with the individual ecosystem services provided by agricultural grasslands (from Dodd et al. 2023).

## Data Availability

The datasets used or analysed during the current study are available in the supplementary information for this manuscript.
