# Supplementary material for: Biochar application to temperate grasslands: challenges and opportunities for delivering multiple ecosystem services
Source: Biochar. 2023 Jun 12;5(1):33. doi: 10.1007/s42773-023-00232-y (PMC10261193; doi:10.1007/s42773-023-00232-y)
Supplement: Supplementary file 2 — Additional file 2: Table S1. Indicator metrics associated with the individual ecosystem services provided by agricultural grasslands (from Dodd et al. 2023). [file 42773_2023_232_MOESM2_ESM.docx]

**Additional information**

**Biochar application to temperate grasslands – challenges and opportunities for delivering multiple ecosystem services**

Robert W. Brown^1*^, David R. Chadwick^1^, Tom Bott^2^, Helen M. West^2^, Paul Wilson^2^, Genevieve R. Hodgins^3^, Colin E. Snape^3^, and Davey L. Jones^1,4^

^1^*School of Natural Sciences, Bangor University, Bangor, Gwynedd, LL57 2UW, UK*

^2^*School of Biosciences, University of Nottingham, Sutton Bonington, Loughborough LE12 5RD, UK*

^3^*Department of Chemical and Environmental Engineering, University of Nottingham, Jubilee Campus, Nottingham NG7 2TU, UK*

^4^*SoilsWest, Centre for Sustainable Farming Systems, Food Futures Institute, Murdoch University, Murdoch, WA, 6150, Australia*

Table S1 - Indicator metrics associated with the individual ecosystem services provided by agricultural grasslands (from Dodd et al., 2023).

| **Environment Service category** | **Service** | **Indicator category** | **Measurement** |
| --- | --- | --- | --- |
| Provisioning | Food | Plant | Biomass, forage quality, plant survey |
|  | Biodiversity | Plant species richness and diversity | Plant survey |
| Regulating | Air quality | Ground cover | % cover, plant diversity? |
|  |  | Pollutants | NH_3_ and CH_4_ emissions |
|  | Climate regulation | GHG emissions | CO_2_, N_2_O, CH_4_ |
|  |  | C storage | Tea-bag index, DOC loss |
|  |  | Microbial community | Microbial biomass, PLFA taxonomic groups |
|  | Water regulation | Ground cover | % cover |
|  |  | Soil structure | Porosity, bulk density, soil moisture |
|  |  | Soil chemistry | pH, EC, extractable elements (N, P, Al, Fe, K, Mg, Mn, Na, Ca), TOC, TN |
|  |  | Macrofauna | Earthworm biomass and abundance |
|  | Erosion regulation | Ground cover | % cover |
|  |  | Soil structure | Porosity, bulk density, soil moisture |
|  | Water purification | Soil structure | Porosity, bulk density, soil moisture |
|  |  | Soil chemistry | pH, EC, mineral N, extractable elements (P, Al, Fe, K, Mg, Mn, Na, Ca) TC, TN |
|  |  | Soil solution | pH, EC, dissolved nutrients (NO_2_-N, NH_4_,N, PO_4_-P) |
|  |  | Microbial community | Microbial biomass, PLFA taxonomic groups |
|  |  | Macrofauna | Earthworm biomass and abundance |
|  | Pollination | Plant community | Plant survey |
|  | Natural hazard regulation | Ground cover | % cover |
| Supporting | Soil formation | Soil structure | Porosity, bulk density, soil moisture, aggregate stability |
|  |  | Macrofauna | Earthworm biomass and abundance |
|  | Primary production | Ground cover | % cover |
|  |  | Plant community | Plant survey |
|  |  | Microbial community | Microbial biomass, PLFA taxonomic groups |
|  |  | Macrofauna | Earthworm biomass and abundance |
|  | Soil fertility | Soil chemistry | pH, EC, extractable elements (N, P, Al, Fe, K, Mg, Mn, Na, Ca), TOC, TN |
|  |  | Microbial community | Microbial biomass, PLFA taxonomic groups |
|  |  | Macrofauna | Earthworm biomass and abundance |
| Cultural | Aesthetic values | Ground cover | % cover |
|  |  | Plant community | Plant surveys |
|  | Social relations | Farm productivity | Biomass |
|  | Ecotourism | Ground cover | % cover |
|  |  | Plant community | Plant surveys |
